# Effect of In Situ Annealing Treatment on the Mobility and Morphology of TIPS-Pentacene-Based Organic Field-Effect Transistors

**DOI:** 10.1186/s11671-017-2238-y

**Published:** 2017-08-23

**Authors:** Fuqiang Yang, Xiaolin Wang, Huidong Fan, Ying Tang, Jianjun Yang, Junsheng Yu

**Affiliations:** 1 State Key Laboratory of Electronic Thin Films and Integrated Devices, School of Optoelectronic Information, University of Electronic Science and Technology of China, Chengdu, 610054 People’s Republic of China; 2 State Key Laboratory of Electronic Thin Films and Integrated Devices Zhongshan Branch Office, College of Electronic and Information Engineering, University of Electronic and Technology of China, Zhongshan Institute, Zhongshan, 528402 China

**Keywords:** Organic field-effect transistors, In situ annealing treatment, Field-effect mobility, Morphology, TIPS-pentacene

## Abstract

In this work, organic field-effect transistors (OFETs) with a bottom gate top contact structure were fabricated by using a spray-coating method, and the influence of in situ annealing treatment on the OFET performance was investigated. Compared to the conventional post-annealing method, the field-effect mobility of OFET with 60 °C in situ annealing treatment was enhanced nearly four times from 0.056 to 0.191 cm^2^/Vs. The surface morphologies and the crystallization of TIPS-pentacene films were characterized by optical microscope, atomic force microscope, and X-ray diffraction. We found that the increased mobility was mainly attributed to the improved crystallization and highly ordered TIPS-pentacene molecules.

## Background

Organic field-effect transistors (OFETs) have attracted considerable attention as a promising candidate for its practical applications in flexible electronic papers, flat-panel displays, radio frequency identification (RFID) tags, and logic circus [1–7]. Up to now, several strategies such as blade coating [6, 8, 9], ink-jet printing [10, 11], gravure printing [12, 13], and the recently emerged spraying technologies [14–16] have been proved to be efficient methods for the fabrication of electronic devices. Among these methods, spray coating has been investigated intensively due to its unique advantage in manufacturing. Through the spray-coating method, various materials with low solubility in less toxic solvent can be applied due to the requirement of a low solution concentration [17]. Moreover, spray coating makes it possible with higher speed of production and better compatibility to various substrates [18], and the different shapes of film can be patterned through shadow masks [19]. Additionally, compared to other methods, such as spin casting, blade coating, and gravure printing, the spray-coating process can realize a continuous film without damaging the bottom layer of the device: just simply control the solvent content, droplet size, and solidification dynamics.

In the previous works, some novel manufacturing methods have been applied to achieve high-performance OFETs via spray coating. Khim et al. investigated the effects of the droplet size on the performance of OFETs fabricated using spray-printed organic semiconducting active layers [16]. Park et al. made an intensive study of solvent content by using a solvent-assisted post-treatment method [20]. Meanwhile, substrate heating is demonstrated to be an effective method in enhancing the crystallinity of semiconductor films [21, 22]. For that, multiple research work has been developed. Sarcletti et al. researched the mutual influence of surface energy and substrate temperature on the mobility in organic semiconductors [23]. Also, Padma et al. investigated the influence of substrate temperature on the growth modes of copper phthalocyanine thin films at the dielectric/semiconductor interface [24]. Subsequently, Mikayelyan et al. studied the effect of the substrate temperature on the structure and morphology of the vacuum-evaporated films [25]. And the thermal annealing effect on the crack development also has been investigated [26]. Although a large number of studies have focused on improving the intrinsic electrical properties of device fabrication techniques, the influence of in situ annealing treatment in the research field of spray-coated OFETs has not received much attention. Meanwhile, the conventional solution process of OFETs usually calls for production interruptions and baking treatment as well as the process being time consuming. Therefore, the development of a novel annealing processing technique is thus a key step towards utilizing the full potential of the spray process.

In this study, we introduced a simple in situ annealing treatment in fabricating high-performance OFETs, and various substrate temperatures were applied in the in situ annealing treatment. With the 60 °C in situ annealing treatment, the mobility of the OFET device significantly enhanced from 0.056 to 0.191 cm^2^/Vs, which was mainly attributed to the improved crystallization and ordered 6,13-bis(triisopropyl-silylethynyl) pentacene (TIPS-pentacene) molecules. To elucidate the mechanism of this performance enhancement, optical microscope, atomic force microscope (AFM), and X-ray diffraction (XRD) were used to analyze the morphology and crystallization of the TIPS-pentacene films. Our work demonstrates that with simple in situ annealing treatment, high-performance OFETs with an efficient manufacturing process can be realized by carefully controlling the conditions of the in situ annealing method.

## Methods

**Fig. 1:**
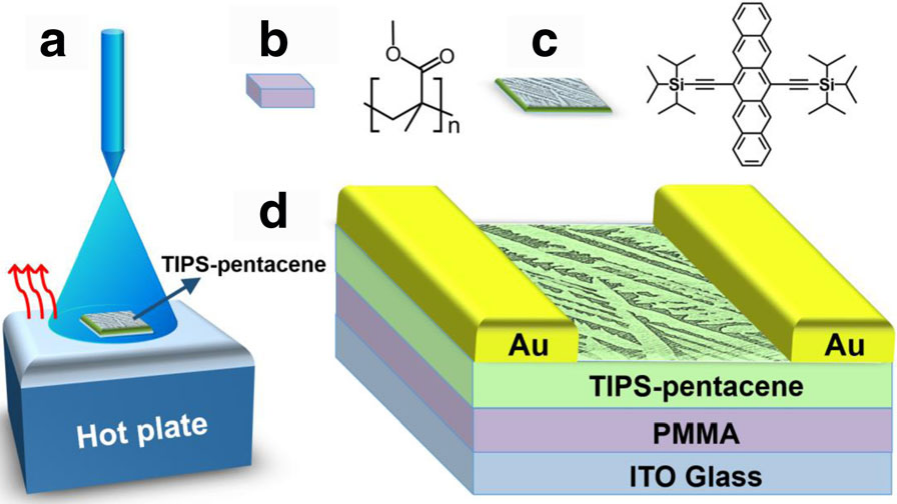
**a** Schematic representation of OFET fabrication by spray coating. **b**, **c** Molecular structures of PMMA and TIPS-pentacene and **d** device architecture of the OFET used in this study

The device fabrication apparatus is shown in Fig. 1(a). The chemical structures of poly(methyl methacrylate) (PMMA) and 6,13-bis(triisopropyl-silylethynyl) pentacene (TIPS-pentacene) are shown in Fig. 1(b) and (c), respectively. The bottom gate top-contacted configuration of OFETs with PMMA dielectric is illustrated in Fig. 1(d). The indium tin oxide (ITO)-coated glasses were used as substrates and gate electrodes. The OFETs were fabricated in the following procedure. Firstly, the ITO glasses placed on a polytetrafluoroethylene (PTFE) holder were ultrasonic cleaned in detergent, acetone, deionized water, and isopropyl alcohol for 15 min each. PMMA was dissolved in anisole with a concentration of 100 mg/mL. Then, a 520-nm PMMA film, functioning as the gate dielectric, was spin coated on the substrates and baked at 150 °C for 1 h in air to remove the solvent residue. Thirdly, the 30-nm TIPS-pentacene active layer was deposited onto substrates placed on a hot plate via a spray-coating process with in situ annealing treatment, and the concentration of the TIPS-pentacene solution was 3 mg/mL in dichlorobenzene. During our experiments, the speed of spray coating was 20 μL/s and the height (from the airbrush to the substrate) was 12 cm, and all the experiments were done at room temperature (20 °C). Finally, a 50-nm-thick gold (Au) was thermally deposited as the source and drain electrodes on the TIPS-pentacene film by a shadow mask. The thickness of the TIPS-pentacene film was characterized by a step profiler. The pure PMMA layer and the PMMA/TIPS-pentacene layer were measured separately, and the thickness of the TIPS-pentacene film can be calculated by subtraction. The device channel width/length ratios are 100 (*L* = 100 μm, *W* = 1 cm). The electrical characteristics of all devices were measured with a Keithley 4200 source meter (Cleveland, OH, USA) in air atmosphere. The field-effect mobility (*μ*) was extracted in the saturation regime from the highest slope of |*I*_DS_|^1/2^ vs. *V*_GS_ plots by using the following equation:

$$ {I}_{\mathrm{DS}}=\left(W/2L\right)\mu {C}_{\mathrm{i}}\left({V}_{\mathrm{GS}}-{V}_{\mathrm{TH}}\right) $$

where *I*_DS_ is the drain-source current, and *L* (100 μm) and *W* (1 cm) are the channel length and width, respectively. *C*_i_ is the capacitance per unit of the dielectric layer, and *V*_GS_ and *V*_TH_ are the gate voltage and the threshold voltage, respectively. The surface morphologies of the TIPS-pentacene were characterized with an optical microscope (U-MSSP4, OLYMPUS) and atomic force microscope (AFM) (MFP-3D-BIO, Asylum Research) in a tapping mode, and the structure characterization was taken by X-ray powder diffraction (XRD, TD-3500, Dandong, China) with an accelerating voltage of 30 kV and an applied current of 20 mA.

## Results and Discussion

**Fig. 2:**
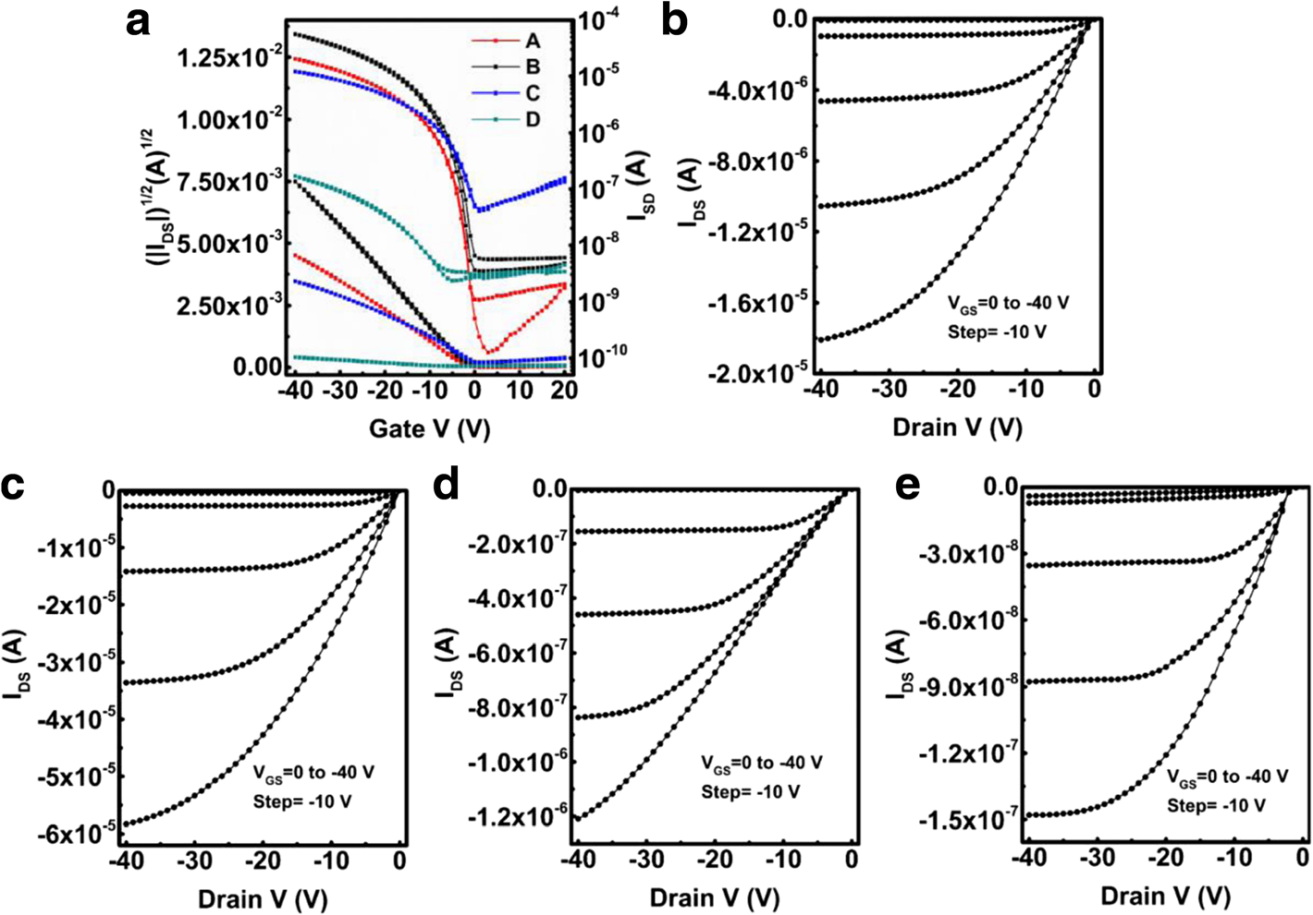
**a** Transfer curves of devices A–D. **b**–**e** Output curves of devices A, B, C, and D, respectively

The OFETs based on 120 °C post-annealing treatment for 20 min were fabricated as device A, and those based on in situ annealing treatment with the temperatures of 60, 90, and 120 °C were fabricated as devices B, C, and D, respectively. The typical transfer characteristic, tested at a source-drain voltage (*V*_DS_) of −40 V and the gate voltage (*V*_GS_) of 20 to −40 V, was tested and presented in Fig. 2a. The output characteristics were tested under a *V*_DS_ of −40 V and a *V*_GS_ of 0 to −40 V at a step of −10 V, as shown in Fig. 2b–e. Several fundamental parameters, including saturation current (*I*_on_), field-effect mobility (*μ*), threshold voltage (*V*_T_), subthreshold swing (SS), and on/off ratio (*I*_on_/*I*_off_), which could be used to evaluate the performance of OFET are summarized in Table 1.

**Table 1:** Electrical characteristics of the OFETs spray coated with post-annealing of 120 °C for 20 min and in situ annealing treatment of 60, 90, and 120 °C, respectively

Annealing temperature	*I*_on_ (10^−6^ A)	*μ* (10^−2^ cm^2^/Vs)	*V*_TH_ (V)	*I*_on_/*I*_off_	SS (V/decade)
Post-annealing	20.31	5.56	−1.7	1.62 × 10^5^	1.40
60 °C (in situ annealing)	76.87	19.05	−0.9	3.47 × 10^4^	1.25
90 °C (in situ annealing)	12.10	4.00	2.0	3.00 × 10^2^	3.81
120 °C (in situ annealing)	0.17	0.05	−3.5	71	4.52

Not unexpectedly, all devices demonstrated typical p-type transistor characteristics. It can be clearly found that the in situ annealing treatment has tremendous influence on the electronic properties of OFETs. Especially, with the 60 °C in situ annealing treatment, the electrical performance of OFET was successfully enhanced, including a positive shift of *V*_TH_ (from −1.7 to −0.9 V), and an increasing *μ* (from 0.056 to 0.191 cm^2^/Vs); the mobility of device B is almost fourfolds higher than that of the post-annealed device A. However, when applying with 90 °C in situ annealing treatment, an extensive degradation of device performance appears along with the increasing substrate temperature, including a forward drift of *V*_TH_ from −0.9 to 2.0 V, and a decreasing *μ* ranged from 0.191 to 0.04 cm^2^/Vs. Furthermore, when the in situ annealing temperature increased to 120 °C, things get even worse, and an obvious decrease of *I*_on_ from 12.1 to 0.17 μA and *μ* from 0.04 to 0.0005 cm^2^/Vs was obtained. As a result, the performance of devices C and D was much worse than the post-annealed device A.

The representative transfer and output plots of the OFETs prepared by spray-coating method with different annealing treatment are depicted in Fig. 2. It can be clearly seen that device B demonstrates the highest electrical performance, including near zero threshold voltages and a narrow subthreshold swing. However, with the increase of substrate temperature in the in situ annealing treatment, an attenuation of electrical performance was revealed. The subthreshold swing exhibited an obvious trend of increment along with the in situ annealing temperature, which implies a relatively high trap density at the interface between the dielectric and semiconductor layer [27].

**Fig. 3:**
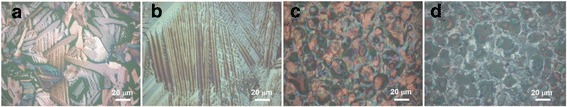
Optical microscope images of spray-coated TIPS-pentacene layer. **a** Substrate temperature of room temperature followed by post-annealing at 120 °C for 20 min, **b**–**d** In situ annealing temperature of 60, 90, and 120 °C, respectively

To scrutinize the surface morphology of TIPS-pentacene films, an optical microscope was employed. As depicted in Fig. 3, the diverse shapes and morphologies of TIPS-pentacene films were obtained, and different crystal grain sizes can be obviously seen from the optical microscope. Large crystal grains are presented in Fig. 3a, b, and the TIPS-pentacene film with the 60 °C in situ annealing treatment is much more uniform, and slender and longish grains are found to grow along the direction of the channel. It indicates a better organization of TIPS-pentacene molecules, resulting in the better electrical performance of the OFET device. However, when the template temperature rises to 90 or 120 °C, circular morphology with small grains start to appear in devices C and D, as shown in Fig. 3c, d. According to the previous study, the alteration of TIPS-pentacene film morphologies would lead to the variation of the electrical properties of OFET devices [28–30].

**Fig. 4:**
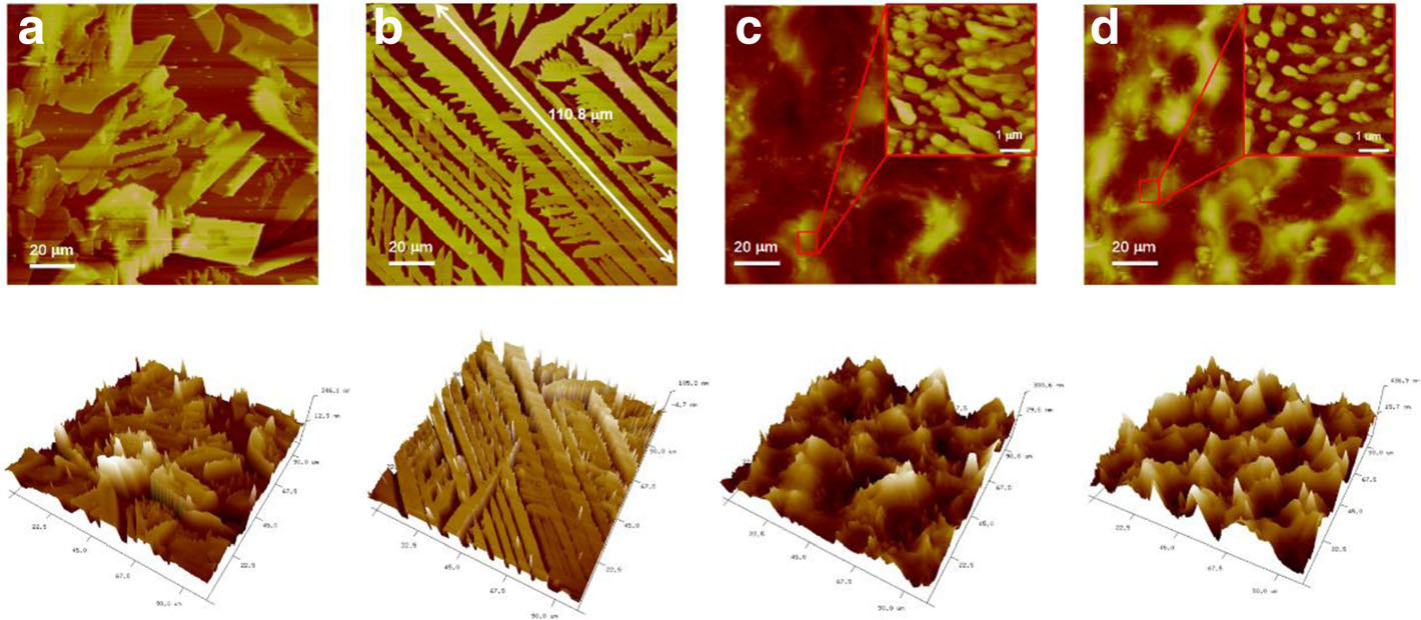
AFM height and 3D images of spray-coated TIPS-pentacene layer. **a** Substrate temperature of RT (followed by post-annealing at 120 °C 20 min). **b**–**d** In situ annealing temperatures of 60, 90, and 120 °C, respectively. *Insets*: high magnification AFM; the scan size bar of inserts is 1 μm

Furthermore, AFM was employed to characterize the morphologies of spray-coated TIPS-pentacene films. As depicted in Fig. 4b, well-ordered TIPS-pentacene grains are formed on PMMA dielectric, whereas irregular crystal grains with different shapes are shown in Fig. 4a, which corresponds well with the optical microscope images in Fig. 3a and b. Interestingly, when the substrate temperature exceeded 60 °C, significant changes in the TIPS-pentacene film morphology can be observed. Figure 4c, d show typical sprayed rounded morphology with a large density of small TIPS-pentacene grains, and these grains exhibit microcrystalline morphology comprising of many island clusters with different sizes as shown in the inserts. Additionally, with further increasing annealing temperature to 120 °C, a much smaller grain array is formed resulting in sparse distribution with plentiful grain boundaries to have a negative effect on carrier transport [16, 31, 32]. Such results indicate that the annealing temperature can greatly affect the film-forming properties, leading to a significant difference in film morphologies.

As we can see, the changes in substrate temperature lead to different morphologies and grain size. And the greatest morphology of device B can be ascribed not only to the proper annealing temperature but also to the favored condition for molecular self-organization. When the OFETs are prepared at a relatively low substrate temperature, gentle evaporation of the solvent can be maintained, leading to a reduced solvent evaporation rate, and the consecutive droplets kept the film wet. Actually, this modulation of substrate temperature directly influences the solvent evaporation rate. Lower annealing temperature allows TIPS-pentacene crystals to grow slowly with ordered molecules [33], while higher substrate temperature contributes to quickly solidification, without a relatively slow drying process of solvent [34]. Thus, a longer time was obtained for molecular self-organization during the spray process, which is responsible for a higher degree of phase separation and a larger domain size [33, 35, 36]. As a consequence, slender and longish grains are formed, and the bridges for carrier transportation in the channel region can be built through these long grains which are longer than 110.8 μm [37].

**Fig. 5:**
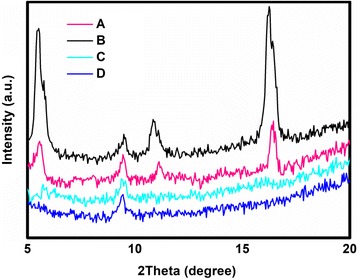
Normalized XRD spectra of spray-coated TIPS-pentacene films with both post-annealing and in situ annealing treatment

To further investigate the molecule orientation and packing in the spray-coated TIPS-pentacene films, XRD was introduced. As shown in Fig. 5, the individual traces exhibit a series of narrow Bragg peaks assignable to the reflections (00*l*) of TIPS-pentacene [38], and the density indicates that the substrate temperature will dramatically affect the crystallinity of the TIPS-pentacene molecules [39]. Compared to device A with post-annealing treatment, device B has the strongest peak intensity, which is consistent with the micrographs of the TIPS-pentacene films, indicating that the TIPS-pentacene deposited with 60 °C in situ annealing treatment yields the best crystallinity of TIPS-pentacene. When the substrate temperature increases to 90 and 120 °C, an inferior order of TIPS-pentacene was formed, which was responsible for the decline in the device performance [40].

## Conclusions

In summary, we have fabricated and tested OFETs by spray coating TIPS-pentacene with in situ annealing treatment, and the surface morphologies and the crystallization of the obtained film were investigated. The results show that the electrical performance of TIPS-pentacene-based OFETs has strong correlation with the processing condition of the active layer. With the template temperature of 60 °C, the mobility of OFETs fabricated by in situ annealing method increases from 0.056 to 0.191 cm^2^/Vs. The performance enhancement was attributed to the higher crystallization and ordered grains. This in situ annealing treatment of the spray-coating method is expected to be an effective way for the fabrication of high-performance OFETs as well as a high potential for low-cost manufacturing and application versatility.

## Authors’ Information

Fuqiang Yang received his B.A. degree from Wuhan Textile University in 2015. He has been studying for his Master degree at State Key Laboratory of Electronic Thin Films and Integrated Devices (SKLETFID) and UESTC since 2015, where his main research interests are in OFETs and OFET-based sensors.

Xiaolin Wang has been studying for his B.A. degree at the School of Optoelectronic Information at UESTC since 2016. He has been working as a student at SKLETFID since 2017, where his main research interests are in OFETs and OFET-based sensors.

Huidong Fan received his B.A. degree from the Yingcai Experimental School at UESTC in 2013. He has been studying for his Ph.D. degree at the State Key Laboratory of Electronic Thin Films and Integrated Devices (SKLETFID) at UESTC since 2013, where his main research interests are in OFETs and OFET-based sensors.

Ying Tang got her Ph.D. degree from the Graduate School of Physical Science and Technology at Lanzhou University in 2007. She is a postdoctor of SKLETFID at UESTC majoring in organic electronic materials and devices.

Jianjun Yang got his Ph.D. degree from UESTC in 2002. Now, he is the professor of the State Key Laboratory of Electronic Thin Films and Integrated Devices Zhongshan Branch Office, College of Electronic and Information Engineering, University of Electronic and Technology of China, Zhongshan Institute, majoring in optoelectronic materials and devices.

Junsheng Yu got his Ph.D. degree from the Graduate School of Bio-Applications and System Engineering at Tokyo University of Agriculture and Technology in 2001. He is the Professor of SKLETFID and UESTC majoring in organic photoelectronic and electronic materials and devices.
